# Expression Improvement of Recombinant Plasmids of the Interleukin-7 Gene in Chitosan-Derived Nanoparticles and Their Elevation of Mice Immunity

**DOI:** 10.3390/biology12050667

**Published:** 2023-04-28

**Authors:** Wenli Hou, Linhan Zhang, Jianlin Chen, Yiren Gu, Xuebin Lv, Xiuyue Zhang, Jiangling Li, Hui Liu, Rong Gao

**Affiliations:** 1 Key Laboratory for Bioresource and Eco-Environment of the Education Ministry, College of Life Sciences, Sichuan University, Chengdu 610064, China; 2 School of Laboratory Medicine, Chengdu Medical College, Chengdu 610500, China; 3 Sichuan Academy of Animal Science, Chengdu 610066, China; 4 R&D Center, Chengdu Kanghua Biological Products Co., Ltd., Chengdu 610100, China

**Keywords:** pig interleukin-7, chitosan derivatives, nanoparticle, gene expression, immunity, mice

## Abstract

**Simple Summary:**

To explore a safe and effective way to potentiate the systemic immunity of animals against infectious diseases in an economical way, the interleukin-7 (IL-7) gene of Tibetan pig was utilized to construct a recombinant eukaryotic plasmid. We first detected the bioactivity of expressed IL-7 on porcine lymphocytes in vitro and then encapsulated the IL-7 gene with different chitosan (CS) and its two modified derivatives, CS-PEG-PEI and CS-PEG-GAL nanoparticles. Then, we injected mice intramuscularly or intraperitoneally with various nanoparticles containing the IL-7 gene to evaluate their immune effects in vivo. We observed a significant increase in neutralizing antibodies and specific IgG levels in response to the rabies vaccine in the treated mice compared to the controls. Treated mice also manifested elevated leukocytes, CD4+ and CD8+ T cells, and mRNA levels of toll-like receptors (TLR1/4/6/9), IL-1, IL-2, IL-4, IL-6, IL-7, IL-23, and transforming growth factor-beta (TGF-β). Notably, the recombinant IL-7 gene encapsulated in CS-PEG-PEI provoked the best increases of immunoglobulins, CD4+ and CD8+ T cells, TLRs, and cytokines in the blood of the mice, implying that chitosan-PEG-PEI can become a promising carrier for in vivo IL-7 gene expression to enhance the innate and adaptive immunity for the prevention of animal diseases.

**Abstract:**

To investigate a safe and effective approach for enhancing the in vivo expression of recombinant genes and improving the systemic immunity of animals against infectious diseases, we employed the interleukin-7 (IL-7) gene from Tibetan pigs to construct a recombinant eukaryotic plasmid (VRTPIL-7). We first examined VRTPIL-7’s bioactivity on porcine lymphocytes in vitro and then encapsulated it with polyethylenimine (PEI), chitosan copolymer (CS), PEG-modified galactosylated chitosan (CS-PEG-GAL) and methoxy poly (ethylene glycol) (PEG) and PEI-modified CS (CS-PEG-PEI) nanoparticles using the ionotropic gelation technique. Next, we intramuscularly or intraperitoneally injected mice with various nanoparticles containing VRTPIL-7 to evaluate their immunoregulatory effects in vivo. We observed a significant increase in neutralizing antibodies and specific IgG levels in response to the rabies vaccine in the treated mice compared to the controls. Treated mice also exhibited increased leukocytes, CD8+ and CD4+ T lymphocytes, and elevated mRNA levels of toll-like receptors (TLR1/4/6/9), IL-1, IL-2, IL-4, IL-6, IL-7, IL-23, and transforming growth factor-beta (TGF-β). Notably, the recombinant IL-7 gene encapsulated in CS-PEG-PEI induced the highest levels of immunoglobulins, CD4+ and CD8+ T cells, TLRs, and cytokines in the mice’s blood, suggesting that chitosan-PEG-PEI may be a promising carrier for in vivo IL-7 gene expression and enhanced innate and adaptive immunity for the prevention of animal diseases.

## 1. Introduction

Interleukin-7 (IL-7) is a key cytokine for adaptive animal immunity, ensuring the growth and differentiation of myeloid and lymphoid cells [1,2,3]. Early research in the 1980s demonstrated its ability to promote pre-B cell growth in bone marrow cultures [4,5]. Recent studies show that IL-7 regulates the differentiation of pre-T cells into various immunocompetent T subpopulations, such as cytotoxic T cells (Tc), helper T cells (Th), and memory T cells (Tm) [6,7]. Furthermore, IL-7 participates in immunity against viral infections and other diseases and plays a role in aging and autoimmunity regulation [8,9]. As a result, IL-7 research has garnered increasing interest in both basic and clinical immunoregulatory fields [10,11].

Tibetan pigs inhabit the Tibetan plateau, characterized by a harsh, hypoxic environment above sea level exceeding 4 km [12]. The IL-7 gene, known to regulate animal immune functions both in vitro and in vivo, has been identified in various species, including Tibetan pigs. Studies have shown that Tibetan pigs possess stronger immunity and greater resistance to infectious diseases such as classical swine fever virus (CSFV), porcine reproductive and respiratory syndrome virus (PRRSV), and porcine circovirus 2 (PCV2) than other domestic or crossbred pigs, likely due to their higher expression levels of β-defensin [13]. Although Tibetan pigs exhibit distinct evolutionary patterns compared to other pigs, the reasons for their enhanced immunity and viability remain unclear. Consequently, we chose RNA extracted from Tibetan pig lymphocytes as our IL-7 gene template (TPIL-7) to investigate its impact on mice immunity.

IL-7’s regulatory effects span the proliferation and differentiation of T cells, B cells, dendritic cells, macrophages, natural killer (NK) T cells, and other immune cells. Additionally, IL-7’s role as a homeostasis mediator may be linked to its regulation of IgG subclasses. Recent research found that recombinant IL-7 substantially promoted lymphocyte development in tumor-bearing animal models, increased central and effector memory CD8 T cells, and downregulated regulatory T cells (Tregs) in tumors, thus enhancing survival [14]. Moreover, IL-7 interacts with its receptor (IL-7R), triggering essential signaling pathways that stimulate early B lymphopoiesis [15]. The differentiation of lymphoid progenitors into pro-B cells was shown to be mediated by IL-7 and IL-7R signaling, initiating immunoglobulin heavy (IgH) chain gene rearrangement, which facilitated the transition from pre-B to pro-B cells, ensuring B cell proliferation [16]. Other studies discovered that IL-7 could increase antibody responses against pathogens, playing a critical role in adaptive immune processes [17]. The production of specific IgG antibodies can be efficiently augmented by IL-7/antigen combinations in vitro via B lymphocyte activation [18].

Chitosan, an N-deacetylated chitin derivative primarily sourced from crustacean exoskeletons, is an innovative biological material extensively utilized in the biomedical industry as a nanoparticle carrier for drug delivery, an immunogen wrapper, and a powerful adjuvant for antigen vaccination [19,20]. Significant evidence supports the reliability of chitosan salts and their derivatives as vaccine adjuvants, exhibiting low local toxicity and effectively enhancing immunogenicity [21]. However, the regulatory effect of chitosan as an immunopotentiator for cytokines in immunological responses remains uncertain.

This study aims to examine IL-7 derived from Tibetan pigs and further investigate its immune characteristics, which hold considerable agricultural economic value in pig breeding, vaccination, and disease prevention. The adjuvant potential of various chitosan derivatives is also explored. In this study, the Tibetan pig IL-7 gene was first cloned, and its impact on mice immunity in vivo was examined. Our findings may offer insights into safe and effective immunoadjuvants to enhance the prevention of animal contagious diseases later.

## 2. Materials and Methods

### 2.1. Pig Lymphoblasts In Vitro

Lymphoblasts were prepared and isolated from the whole blood of a Tibetan pig (reared in Sichuan Academy of Animal Science) using Ficoll-Paque PREMIUM (1.077 g/mL, Cytiva, Shanghai, China) and cultured in a complete 1640 medium containing 5 ug/mL concanavalin A (Con A) at 37 °C with 5% CO_2_ for 2 days. Then, the RNA was isolated from the provoked lymphocytes using an RNA extraction kit (Invitrogen, Carlsad, CA, USA) and prepared for further TPIL-7 gene cloning

### 2.2. Cloning & Sequencing of TPIL-7 Gene 

The TPIL-7 gene was cloned using RT-PCR and inserted into the pMD^®^19-T vector (Takara, Dalian, China), which was transformed into DH5α competent cells. Its primers were decided using Primer 5.0, and *BamH* I and *Bgl* II sites were chosen based on the conserved IL-7 ORF sequence of Duroc and Landrace in GenBank. TPIL-7-F: 5′-ACCACGCCCGCCTCCCGCAGACCATGTTC-3′, TPIL-7-R: 5′-GAATATTGAGAGATACGGAGTGGCAA-3′. Recombinant plasmids were initially analyzed through PCR and digestion with *BamH* I and *Bgl* II. Then, they were delivered to sequence in BGI biological company. Its sequence was compared with other animals using NCBI/Blast. The TPIL-7 gene was subcloned into the VR1020 eukaryotic expression plasmid (Vical Company, San Diego, CA, USA) to construct VTPIL-7, which expresses a secretory protein for bioactivity evaluation in vitro and in vivo, as described above. 

### 2.3. Construction of VTPIL-7 & Preparation of Plasmid Nanoparticles 

The recombinant DH5a *E. coli* carrying VTPIL-7 or VR1020 was cultured in routine LB broth containing kanamycin (100 mg/mL) in a shaker at 37 °C, 200 rpm for 12 h. They were then centrifuged, and the plasmids were isolated using the E.Z.N.A. ^®^ Plasmid Mini Kit I (OMEGA Bio-Tek, Guangzhou, China). The plasmids were resuspended in pure water and kept at –20 °C.

PEI/DNA complexes, Chitosan copolymer, CS-PEG-LAC and CS-PEG-PEI were prepared using the ionotropic gelation technique [22,23]. In short, CS, CS-PEG-LAC and CS-PEG-PEI were respectively diluted with CH_3_COOH/CH_3_COONa (pH 5.5) fluid at a suitable ratio of triphosphate and heated to 65 °C for 10 min under slight magnetic mixing. A suitable quantity of chitosan solution was then mixed with the plasmid DNA solution, and the solutions were stirred well for 5 min. The nanoparticles were analyzed using Zetasizer 3000 HS/IHPL (Malvern Instruments Ltd., Malvern, UK).

### 2.4. Transfection, Expression Identification & Bioactivity Analysis of TPIL-7 Gene

HEK293 cells were seeded in 12-well plates (1.0×10^5^ cells/well) and incubated in complete Dulbecco’s modified Eagle medium (DMEM, Invitrogen Corporation, Shanghai, China.) containing 10% Fetal Bovine Serum (FBS). The nanoparticles containing the 3 μg of plasmids packed with PEI, CS, CS-PEG-LAC and CS-PEG-PEI were respectively added to the wells, and the cells were cultured at 37 °C in a 5% CO_2_ humidified atmosphere for 48 and 72 h. The transfected cells were collected, and the total RNA was extracted at 48 h to analyze the mRNA expression via RT-PCR. The primers for RT-PCR were designed as previously mentioned.

The TPIL-7 bioactivity was evaluated by measuring the increase of porcine lymphoblasts provoked with Concanavalin A (Con A) using the Cell Counting Kit 8 (CCK8; Dojindo Laboratories, Kumamoto, Japan) colorimetry. In total, 8 treatments, including empty plasmid groups and TPIL-7-containing groups, were measured, and 3 duplicate wells were set up for each sample. Mononuclear immune cells were isolated from peripheral pig blood using a lymphocyte Separation Medium to test their bioactivity in vitro. 

### 2.5. Mice Inoculation 

In this study, 40 female Kunming mice at 4 weeks of age were supplied by the Medical Sciences Animal Center of West China, Sichuan University. The mice were arbitrarily assigned to the C, A1, A2, and A3 groups, with 10 mice per group. On day 0, each mouse received an intramuscular injection into both of left and right quadriceps at the dose of 100 μg plasmid in nanoparticles. Group A1 mice received the injection of CS/VTPIL-7, A2 for CS-PEI-PEG/VTPIL-7, A3 for CS-PEI-LAC/VTPIL-7, and group C for CS-VR1020 as a blank mock. At days 0, 7, 14, 21, 28, and 35 post inoculation, their blood was harvested from their tail vein. The mice were reared in the same conditions for 5 weeks. The animal experiment was approved by the Animal Ethics Committee (AEC) of the Animal Experiment Center of Sichuan University (SYXK-Chuan-2019-147), and all the mice were treated in compliance with Chinese animal welfare laws and regulations.

### 2.6. Bioactivity Analysis of TPIL-7 In Vivo

#### 2.6.1. Quantity of Blood Cells

The blood samples were collected and anticoagulated with EDTA (blood sample: EDTA = 1:10). The samples were double-diluted with normal saline and assayed using the intelligent automatic blood cell analyzer, MIND-RAY BC-3000, which measures erythrocytes, leukocytes, hemoglobin and platelets.

#### 2.6.2. Analysis of Immunocompetent T Cells by Flow Cytometry (FCM)

Monoclonal antibodies for FCM, e.g., Anti-Mouse CD4 PerCP-Cy5.5 (0.25 μg/test), Anti-Mouse CD8a PE (0.25 μg/test), and Anti-Mouse CD3e FITC (0.5 μg/test; eBioscience Company, San Diego, CA, USA) were used. Each test used 50 μL of blood, and dead cells were excluded using Fixable Viability Stain 510 from BD HorizonTM (BD Life Sciences, San Jose, CA, USA). FCM analysis was performed using FACSAria BD and FACSDiVa BD flow cytometers from BD Biosciences (BD Biosciences, San Jose, CA, USA).

#### 2.6.3. Assay of Mouse IgG1, IgG2a and IgG

The IgG1, IgG2a, and IgG levels were quantified in mouse plasma samples from each treatment using mouse IgG, IgG1, and IgG2a measurement ELISA kits following the manufacturer’s protocols (Bethyl Laboratory, Inc., Montgomery, TX, USA).

#### 2.6.4. Transcription Analysis of Cytokines & TLRs Gene

The mRNA levels of IL-23, IL-7, IL-6, IL-4, IL-2, IL-1, TGF-β, TLR9, TLR6, TLR4, TLR1 and β-actin genes of the experimental mice were quantitatively analyzed using RT-qPCR. Specific primers were synthesized based on data in GenBank. The RNA was isolated from the blood of the mice using a Quantscript RT kit from TianGen Biotech (Beijing, China) following the manufacturer’s directions, and cDNAs were synthesized as instructed in the RNA extraction/cDNA synthesis kit manual of Invitrogen. β-actin was used as the reference gene for the RT-qPCR, and the mRNA expression of TGF-β, TLRs and ILs genes were measured. The relative mRNA expressions were computed via the geometric means method with the routine equation: relative level = 2^−ΔΔCt^; ΔΔCt = ΔCt (treated sample) – ΔCt (control sample); ∆Ct = Ct (target gene) – Ct (reference gene). The primers for the q-PCR assay are listed in Table 1.

### 2.7. Effect of TPIL-7 on Rabies Vaccination

#### 2.7.1. Animal and Vaccination

In this experiment, 20 healthy female Kunming mice (16–18 g) at 6 weeks of age were obtained from the Laboratory animal center of Sichuan University and randomly divided into 2 groups: control group (C) and treatment group (T). The mice were intraperitoneally immunized as follows: C group: 7 IU Human diploid cell inactivated Rabies virus vaccine (RABV) from Chengdu Kanghua Biological Products Co., Ltd., Chengdu, China + 100.0 µg CS-PEI-PEG/VR1020; T group: 100.0 µg CS-PEI-PEG/VTPIL-7 + 7 IU RABV vaccine. Booster immunization was carried out using the same method on the 14th day after the first immunization. Blood samples (100 µL) were collected weekly from the tail veins of mice for 49 days. The mice were reared under the same condition. The animal experiment was approved by the Animal Ethics Committee (ACE) of the Animal Experiment Center of Sichuan University (SYXK-Chuan-2021-172).

#### 2.7.2. ELISA Assay of Specific IgG for RABV 

0.5 µg RABV glycoprotein from the State Key Lab of Biotherapy (Sichuan University) was utilized to coat ELISA plates in the pH 8.2 buffer overnight at 4 °C. The plates were then cleaned thrice in PBS-tween (PBST) and blocked with 5 % low-fat milk in PBS for 2 h at 37 °C. The plasma samples were diluted 1:30 in 100 μL PBS and then transferred to the wells containing RABV G. The samples were incubated for 2 h at 37 °C. After incubation, the plates were washed 3 times with PBST solution, and the secondary goat anti-mouse IgG conjugated with HRP (1:10,000; Abcam, Waltham, MA, USA) was put into the wells, which were incubated at 37 °C for 45 min. After incubation, the plates were washed again as before, and then the substrate with tetra-methyl-benzidine (TMB) was employed in the wells as per the manufacturer’s instructions (Mei5 Biotechnology, Co., Ltd., Beijing, China). The reaction was terminated by the addition of 2M H2SO4 in the dark after 15 min incubation at 37 °C. The OD_450_ was read using an M 680 (Bio-Rad, Hercules, CA, USA).

#### 2.7.3. Virus Neutralizing Antibody (VNA) Titers Measurement 

The VNA titers of mice plasma were detected via the fluorescent-antibody virus neutralization (FAVN) assay. Sample plasma and standard sera were diluted 3-fold in a total volume of 100 μL of cell culture medium in 96-well plates. The sample was added to 4 adjacent wells, and 100 FFU of the CVS-11 rabies virus suspended in a 50-μL solution was added to each sample well and incubated at 37 °C for 1 h. Then 50 μL 4 × 10^5^ BHK-21 cells/mL were put into each well and cultured at 37 °C for 72 h. The tested cells were first fixed with 80% ice-cold acetone and then stained with FITC-conjugated antibodies to RABV N protein. The fluorescence was read via an Olympus IX51 fluorescence microscope (Olympus, Tokyo, Japan). The sample fluorescence values were compared to the reference serum values from Changchun Veterinary Research Institute, Changchun, China, and the data were normalized and calculated as international units per milliliter.

### 2.8. Statistical Analysis 

The data were analyzed using an ANOVA test, and differences among experimental groups were considered significant if the *p*-value was less than 0.05 and *vice versa*.

## 3. Results

### 3.1. Cloning and Sequencing TPIL-7 Gene 

The cDNA of the TPIL-7 gene is 571 bp, in which an open reading frame (ORF) is 528 bp, encoding 176 amino acids. When aligned with other pig IL-7 cDNA in NCBI, the homology of TPIL-7 cDNA is over 92%. The sequence has been submitted to GenBank with the ID gb|KF246514.1|.

### 3.2. Nanoparticles Sizes and Zeta Potential 

**Figure 1:**
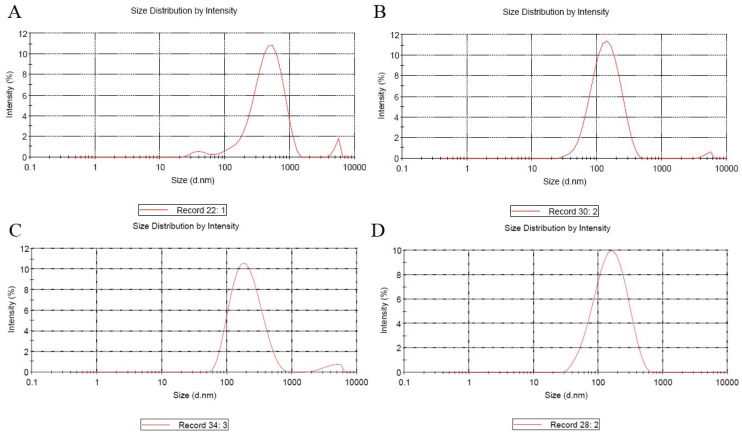
The map of entrapped VTPIL-7 nanoparticles size. (**A**) VTPIL-7-CS; (**B**) VTPIL-7-PEI; (**C**) VTPIL-7-CS-PEG-Lac; (**D**) VTPIL-7-CS-PEI-PEG.

**Figure 2:**
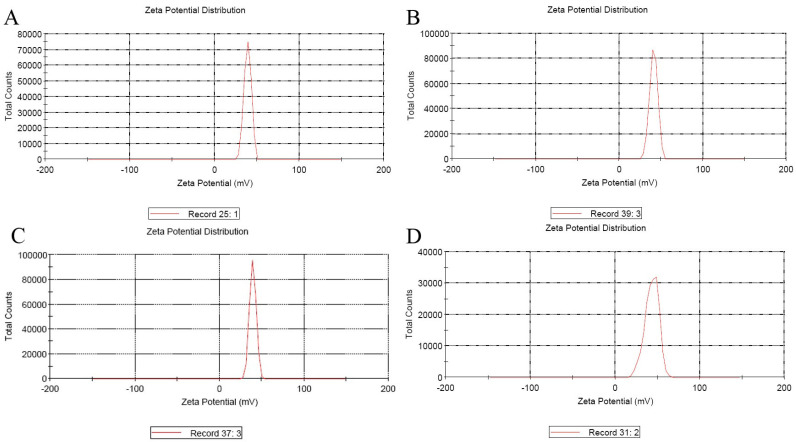
The map of entrapped VTPIL-7 nanoparticles Zeta Potential (mV). (**A**) VTPIL-7-CS; (**B**) VTPIL-7-PEI; (**C**) VTPIL-7-CS-PEG-Lac; (**D**) VTPIL-7-CS-PEI-PEG.

Cationic molecules are excellent candidates for delivering genes into cells, and the suitable diameter and positive surface charge of nanoparticles are essential for cell endocytosis. The size and zeta potentials of different plasmids nanoparticles are recorded in Table 2 and described in Figure 1 and Figure 2. 

### 3.3. Transcription Expression and In Vitro Proliferation of Pig Lymphoblasts 

**Figure 3:**
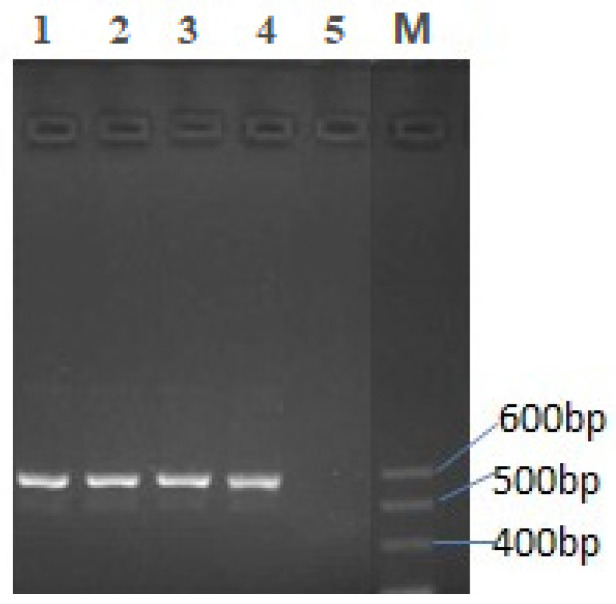
Electrophoresis of RT-PCR products of the samples of cells transfected with different nanoparticles (2% agarose gel). Lane1: RT-PCR product of VTPIL-7-CS, Lane2: RT-PCR product of VTPIL-7-PEI, Lane3: RT-PCR product of VTPIL-7-CS-PEG-Lac, Lane 4: RT-PCR product of VTPIL-7-CS-PEI-PEG, Lane 5: RT-PCR product of VR1020-CS, M: DNA Marker.

The mRNA transcription of the recombinant TPIL-7 gene was analyzed by RT-PCR via agarose gel electrophoresis (Figure 3). The results showed that the four samples of treated cells transfected with different recombinant plasmid nanoparticles manifested a single DNA fragment on the gel, with an expected size of about 570 bp, which is close to the 600 bp band of the marker DNA. In contrast, the control cells transfected with VR1020-CS nanoparticles did not show any product of RT-PCR resulting from IL-7 mRNA (lane 5 of Figure 3). These results confirm that TPIL-7 mRNA was successfully transcribed in the HEK293 cells transfected with the recombinant plasmids packed with chitosan and its derivative nanoparticles. 

**Figure 4:**
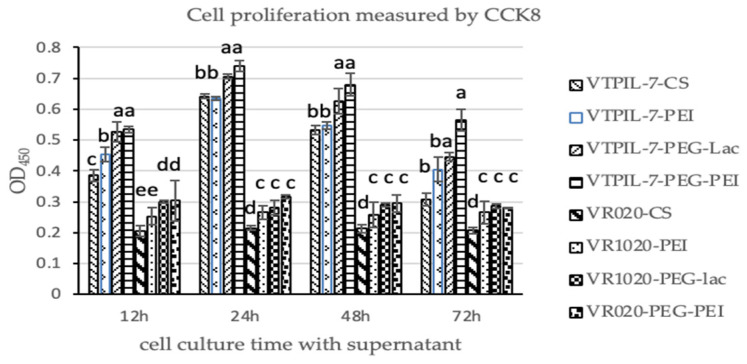
The lymphoblasts proliferation analyzed using a Cell Counting Kit-8 (CCK8) 12–72 h post-inoculation by the supernatant of HEK-293 cells transfected by chitosan-wrapped VTPIL-7s and chitosan-blank plasmid controls. Statistical significance was shown by letter symbols. Different letters above the bars of the groups indicate significant differences at *p* < 0.05. The same letters notated above the bars indicate that the differences among the groups were not significant. The followings are all the same as here.

The results showed that compared to the four blank control groups, the supernatant of TPIL-7 gene-treated cell culture induced the significant proliferation of pig lymphoblasts (Figure 4). Supernatants of CS-PEG-LAC and CS-PEG-PEI achieved the best proliferation, implying that the recombinant plasmids successfully expressed TPIL-7 in the four transfected cells. Additionally, more IL-7 was expressed by the recombinant plasmid packed with the two nanoparticles, which stimulated the remarkable proliferation of lymphocytes in comparison with other groups (*p* < 0.05).

### 3.4. Elevation of Mice Immunity

#### 3.4.1. Immune Changes in the Blood

**Figure 5:**
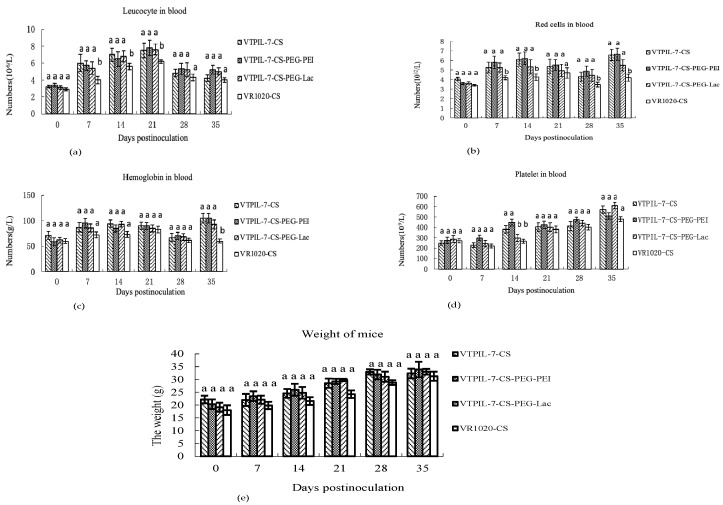
The changes of leukocytes (**a**), erythrocytes (**b**), platelets (**c**), and hemoglobin (**d**) in the peripheral blood and weight (**e**) of VTPIL-7-CS, VTPIL-7-CS-PEG-PEI and VTPIL-7-CS-PEG-Lac treated mice and control groups. Statistical significance was shown by letter symbols. Different letters above the bars of the groups indicate significant differences at *p* < 0.05. The same letters notated above the bars indicate that the differences among the groups were not significant.

Over a 35-day observation period, the leukocytes, erythrocytes, hemoglobin, and platelets of the treated mice significantly increased at different time points compared to the control group (*p* < 0.05). However, the mice treated with different VTPIL-7 nanoparticles did not show significant differences (*p* > 0.05; Figure 5a–d). The weight gain of the injected mice was slightly higher than the control mice (*p* > 0.05; Figure 5e).

#### 3.4.2. Change of CD4+ and CD8+ T Lymphocytes

**Figure 6:**
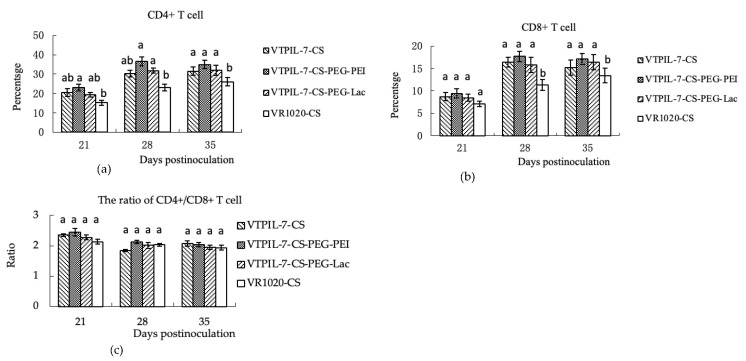
The variation of CD4+ and CD8+ T cells and their ratio in peripheral blood of mice treated with VTPIL-7-CS, VTPIL-7-CS-PEG-PEI, VTPIL-7-CS-PEG-Lac, and plasmid controls. (**a**) CD4+ T cell level (%); (**b**) CD8+ T cell level (%); (**c**) The ratio of CD4+/CD8+ T cell. Statistical significance was shown by letter symbols. Different letters above the bars of the groups indicate significant differences at *p* < 0.05. The same letters notated above the bars indicate that the differences among the groups were not significant.

In comparison with the control mice, both CD4+ and CD8+ T cells significantly increased in the blood of the treated mice from 21 to 35 days after inoculation (*p* < 0.05). The VTPIL-7 in modified chitosan provoked a slightly greater increase of CD4+ and CD8+ T cells than in other treated mice (*p* > 0.05; Figure 6a–c).

#### 3.4.3. Change of IgG1, IgG2a and IgG

**Figure 7:**
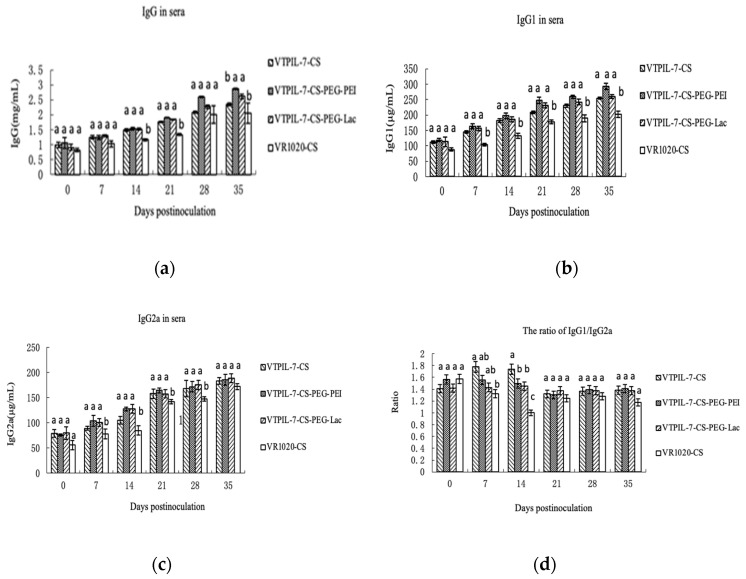
The IgG1, IgG2a, IgG, and their ratio in the sera of mice inoculated with VTPIL-7-CS, VTPIL-7-CS-PEG-PEI, VTPIL-7-CS-PEG-Lac, and plasmid controls was quantified by ELISA. (**a**) IgG level; (**b**) IgG1 level; (**c**) IgG2a level; (**d**) The ratio of IgG1/IgG2a. Statistical significance was shown by letter symbols. Different letters above the bars of the groups indicate significant differences at *p* < 0.05. The same letters notated above the bars indicate that the differences among the groups were not significant.

The levels of IgG1, IgG2a and IgG increased significantly in the sera of the treated mice compared to the control group (*p* < 0.05). The ratio of IgG1/IgG2a remained at about 1.2, suggesting that humoral immunity was significantly enhanced in the treated mice (Figure 7a–d).

#### 3.4.4. Change of TLRs Gene Expression

**Figure 8:**
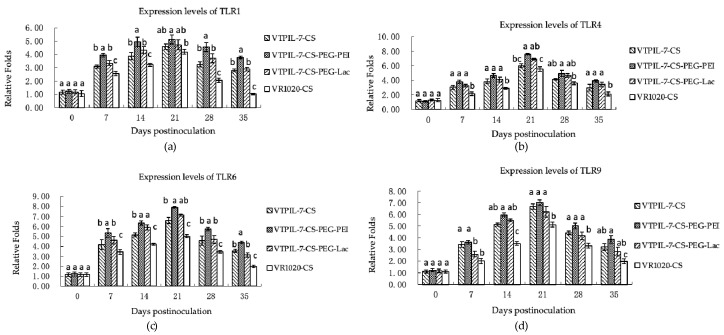
The expression levels of four toll-like receptors (TLRs) at the gene level in inoculated mice analyzed by RT-qPCR. (**a**) The expression level of TLR1; (**b**) The expression level of TLR4; (**c**) The expression level of TLR6; (**d**) The expression level of TLR9. Statistical significance was shown by letter symbols. Different letters above the bars of the groups indicate significant differences at *p* < 0.05. The same letters notated above the bars indicate that the differences among them were not significant.

Figure 8a–d showed that in comparison with the control mice, the expression levels of the TLR1, 4, 6 and 9 genes increased markedly in the treated mice from 7 to 35 days (*p* < 0.05). TLR6 showed a strong promotion effect, which was six times higher before inoculation. The CS-PEG-PEI mice manifested a stronger enhancement effect on TLRs expression than other treated mice (Figure 8a–d).

#### 3.4.5. Expression Increase of Cytokine Genes

**Figure 9:**
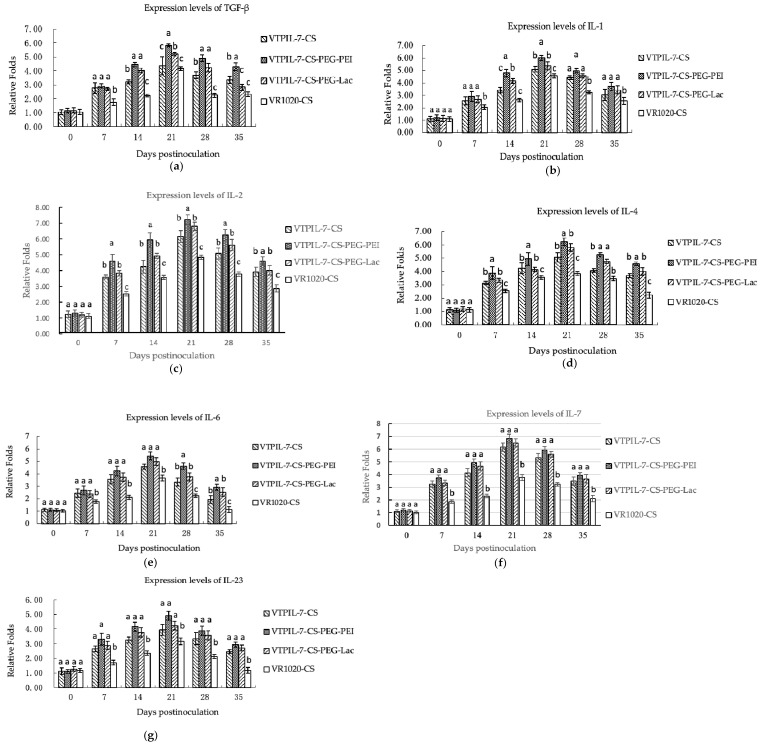
The expression levels of immunoregulatory cytokines at the gene level in inoculated mice analyzed by RT-qPCR. (**a**) TGF-β expression level; (**b**) IL-1 expression levels; (**c**) IL-2 expression level; (**d**) IL-4 expression level; (**e**) IL-6 expression level; (**f**) IL-7 expression level; (**g**) IL-23 expression level. Statistical significance was shown by letter displays. Different letters above the bars indicate significant differences at *p* < 0.05. The same letters notated above the bars indicate that the differences were not significant.

Following the administration of recombinant IL-7 gene nanoparticles, there was a significant increase in the transcription levels of TGF-β, IL-1, IL-2, IL-4, IL-6, IL-7, and IL-23 genes in the blood of treated mice compared to the control group over a period of 35 days (*p* < 0.05). Notably, the CS-PEG-PEI group exhibited significantly better elevations of these cytokine gene expressions in comparison with other treated mice (*p* < 0.05), as depicted in Figure 9a–g.

### 3.5. Regulation on Specific Humoral Immunity to Rabies Vaccination

**Figure 10:**
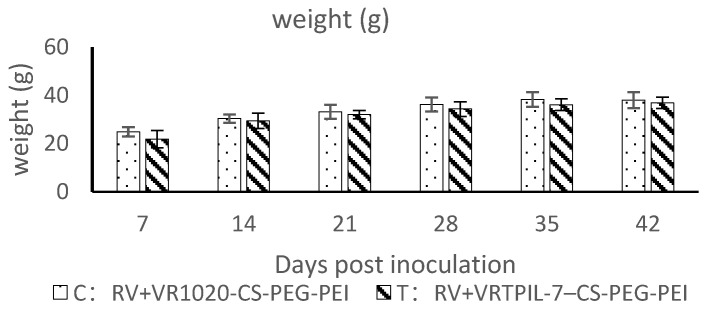
The changes in weights of the experimental mice after Rabie vaccination (*n* = 10).

**Figure 11:**
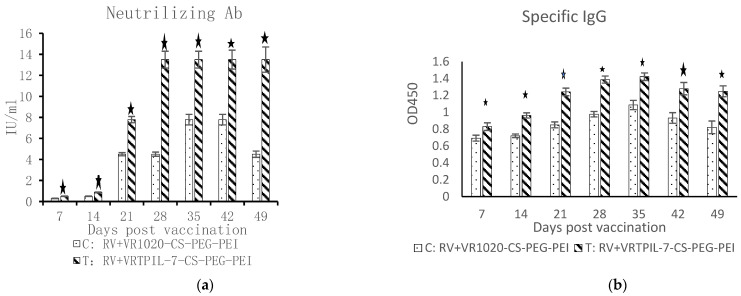
The changes of neutralizing antibodies (**a**) and specific IgG (**b**) in the sera of the experimental mice after Rabie vaccination (*n *= 10). ^★^: *p* < 0.05.

The weight of the treated mice did not show any significant difference from the control mice throughout the observation period after the inoculation with the rabies vaccine (Figure 10; *p* < 0.05). However, the treated mice exhibited a significant increase in neutralizing antibodies in their sera compared to the control mice (Figure 11a; *p* < 0.05). Besides, the specific IgG against Rabies antigen also significantly increased in the treated mice in comparison with that of the control mice (Figure 11b; *p* < 0.05). These results indicate that the specific humoral immune responses of the treated mice were considerably improved by VTPIL-7 in CS-PEI-PEG nanoparticles, which could express the recombinant IL-7 in vivo.

## 4. Discussion

### 4.1. Nanoparticles for Gene Delivery

In this study, chitosan and its derivatives were used as carriers for the delivery of the TPIL-7 gene into cells. The positive zeta potential of nanoparticles is known to be critical for the efficient transfection of recombinant plasmids into cells, and the suitable particle size range is between 30–400 nm [23]. The results of our study demonstrated that the TPIL-7 gene expression was successful, and the supernatant of transfected cells showed significant immune activity in inducing the proliferation of pig lymphoblasts in vitro. This suggests that chitosan and its derivatives nanoparticles can guarantee the success of IL-7 gene expression in eukaryotic cells and that the expressed IL-7 can exhibit proper bioactivity on activated immune cells.

### 4.2. The Role of Chitosan and Its Derivatives in Promoting Immunity

Cationic polymers such as chitosan and PEI have been widely investigated as candidates for gene delivery. Past research revealed that most proteins and DNA complexes easily bond with cationic delivery molecules. Subsequently, multi-cationic PEI is linked to chitosan to raise the positive charge of nanoparticles. The diameter of CS-PEG-PEI was smaller than those packed with chitosan nanoparticles, which was attributed to the elevation of DNA binding/condensation level [24,25]. In our study, CS-PEG-PEI nanoparticles had a smaller average diameter and a higher zeta potential than CS nanoparticles due to the presence of polymeric PEI, which was 43.9 mV and higher than CS nanoparticles due to the existence of polymeric PEI. Consequently, the CS-PEG-PEI elevated the higher mRNA levels of the TGF-β, TLR9, TLR6, TLR4, TLR1, IL-23, IL-7, IL-6, IL-4, and IL-2 genes in the treated mice, which were probably resulted from the complicated interactions of cytokine network. The detailed molecular mechanisms of these interactions are still unclear and require further exploration.

### 4.3. Enhancement of Systemic and Adaptive Immunity by IL-7 Gene Delivery

Previous studies have indicated that recombinant IL-7 can induce high levels of IgG, IgG2a, and IgG2b in vivo and can serve as a promising adjuvant to promote stronger immune responses [26]. TLRs have been found to be the critical regulators involved in antibody production [27]. The deficiency of TLR7 resulted in a reduction of antibody level, while TLR9 signaling activated autoreactive B cells in vitro [28,29]. TLRs and key interleukins (IL-2, IL-6, IL-15 and IL-7 etc.) are essential mediators of both innate and acquired immune responses by signaling on inflammation pathways to defense invading pathogens [30,31]. Similarly, IL-7 plays a critical role in expanding the differentiated T cells to promote specific cellular immunity [32], and the availability of IL-7 is decisive for the survival and homeostasis of peripheral invariant Natural killer T cells (iNKT cells), which is critical for innate and adaptive immunity to resist against microbial infection and inflammation [32]. In fact, Natural killer (NK) T cells are usually positioned at the interface of the innate and adaptive immune systems [33]. iNKT cells can recognize specific antigens and trigger prompt and strong cytokine reactions, and then motivate a series of cascade amplification of defense immunity against microbial infection [34,35].

In our study, the marked expression increases of innate TLRs genes (TLR1/4/6/9) and cytokine genes (IL/1/2/4/6/7/23 and TGF-β) in CS-PEG-PEI group confirmed this beneficial effect on systemic immunity, which were probably resulted from the higher expression of the recombinant IL-7 gene in CS-PEG-PEI nanoparticles. The neutralizing antibody and specific IgG levels to Rabies virus in the serum of mice treated with chitosan-packed VPIL-7 were significantly increased, suggesting that the IL-7 gene delivered with chitosan derivatives had enhanced the humoral immunity of mice. Additionally, the FCM analysis indicated that the CD4+ and CD8+ T lymphocytes significantly increased after injection with chitosan-packed VPIL-7, indicating that IL-7 gene nanoparticles also potentially promoted the proliferation of Tc and Th cells in mice. However, the detailed immune mechanism is still not clear for this immune potentiation by IL-7 expressing plasmid, which needs to be clarified by more explorations later.

Besides, higher levels of CD4+/CD8+ T cells were observed in TPIL-7 treated mice compared to the empty plasmid control group, suggesting that the induction of stronger immunity resulted from PIL-7 expressed by the recombinant plasmid in mice, which shares over 66% identity with murine IL-7 and could similarly regulate cellular immunity of mice.

### 4.4. Safety and Growth Performance of Animals Treated with Recombinant IL-7 Gene

Interestingly, we found that all of the IL-7 recombinant plasmid-treated mice did not show any systematic symptoms or local lesions after inoculation with chitosan and its derivatives nanoparticles. Moreover, their weight gains were higher than the control mice at 21 days after inoculation. Among the IL-7 treated groups, mice with CS-PEG-PEI manifested better growth performance that matched with the stronger immune reactions. This suggests that the delivery of recombinant IL-7 gene by chitosan nanoparticles is safe and has beneficial effects on animal growth. The complicated mechanism between growth and immunity requires further investigation.

Overall, our results suggest that VTPIL-7 packed with chitosan derivatives nanoparticles can significantly promote innate immunity, and humoral and cellular immunity, potentially resulting in vigorous immune defense and disease resistance of animals.

## 5. Conclusions

In conclusion, our study demonstrates that the TPIL-7 gene can be successfully delivered by CS-PEG-PEI nano-particles to promote immune responses in vivo. We observed significant increases in the expression of TLRs and cytokine genes, as well as elevations in leukocytes, immunoglobulins, specific antibodies, and CD4+ and CD8+ T cells in the inoculated mice. Our findings suggest that CS-PEG-PEI packaged TPIL-7 gene has the potential to improve the innate, humoral and cellular immunity of animals and could be further developed as a safe and potent immunobiological molecule for preventing infectious diseases in animals.

## Figures and Tables

**Table 1:** The primers for real-time q-PCR.

Genes	Primers (5′−3′)
β-actin-F	TACGCCAACACGGTGCTGTC
β-actin-R	GTACTCCTGCTTGCTGATCCACAT
TLR-1-F	GGACCTACCCTTGCAAACAA
TLR-1-R	GGTGGCACAAGATCACCTTT
TLR4-F	ACCTGGCTGGTTTACACGTC
TLR-4-R	CTGCCAGAGACATTGCAGAA
TLR-6-F	CCAAGAACAAAAGCCCTGAG
TLR-6-R	TGTTTTGCAACCGATTGTGT
TLR9-F	ACTGAGCACCCCTGCTTCTA
TLR9-R	AGATTAGTCAGCGGCAGGAA
TGF-β-F	GGGAGTAGACAAGGTACAAC
TGF-β-R	ACACACAGCCTCAGTT
IL-1b-F	TGCTGTCGGACCCAT
IL-1b-R	TGTGCCGTCTTTCATTAC
IL-2F	AAGCACAGCAGCAGCAGCAG
IL-2R	GCCGCAGAGGTCCAAGTTCATC
IL-4F	GCCATATCCACGGATGCGACAA
IL-4R	GGTGTTCTTCGTTGCTGTGAGGA
IL-6F	TCTTGGGACTGATGCTGGTGACA
IL-6R	AGCCTCCGACTTGTGAAGTGGTAT
IL-7F	TCCCGCAGACCATGTTCCATGTTTC
IL-7R	TTCAACTTGCGAGCAGCACGA
IL-23-F	TGCTGGATTGCAGAGCAGTAA
IL-23-R	GCATGCAGAGATTCCGAGAGA

**Table 2:** The results of nanoparticles package. Note: SD, Standard Deviation.

Sample	T (°C)	*w/w*	Size (d.nm)		Zeta Potential (mV)	
			Average	SD (±)	Average	SD (±)
**CS/DNA**	**25**	30:1	522.2	5.15	+40.57	1.6
PEI/DNA	25	30:1	341	5.79	+45.5	2.04
CS-PEG-PEI/DNA	25	10:1	169.73	4.072	+43.9	1.113
CS-PEG-LAC/DNA	25	10:1	247.07	18.78	+40.23	0.78

## Data Availability

Not applicable.
